# Imaging Spectrum of Intrahepatic Mass-Forming Cholangiocarcinoma and Its Mimickers: How to Differentiate Them Using MRI

**DOI:** 10.3390/curroncol29020061

**Published:** 2022-01-30

**Authors:** Jelena Djokic Kovač, Aleksandra Janković, Aleksandra Đikić-Rom, Nikica Grubor, Andrija Antić, Vladimir Dugalić

**Affiliations:** 1 Center for Radiology and Magnetic Resonance Imaging, University Clinical Centre of Serbia, Pasterova No. 2, 11000 Belgrade, Serbia; jankovicm.alex@gmail.com; 2 Faculty of Medicine, University of Belgrade, Dr Subotica No. 8, 11000 Belgrade, Serbia; gruborn13@gmail.com (N.G.); drandrija.antic@gmail.com (A.A.); vanjadug@gmail.com (V.D.); 3 Department of Pathology, University Clinical Centre of Serbia, Pasterova No.2, 11000 Belgrade, Serbia; aleksandra.djikic.rom@gmail.com; 4 Clinic for Digestive Surgery, University Clinical Centre of Serbia, Koste Todorovica Street, No. 6, 11000 Belgrade, Serbia

**Keywords:** mass-forming cholangiocarcinoma, mimickers, magnetic resonance imaging

## Abstract

Intrahepatic cholangiocarcinoma (ICC) is the second most common primary hepatic malignancy, with mass-forming growth pattern being the most common. The typical imaging appearance of mass-forming ICC (mICC) consists of irregular ring enhancement in the arterial phase followed by the progressive central enhancement on portal venous and delayed phases. However, atypical imaging presentation in the form of hypervascular mICC might also be seen, which can be attributed to distinct pathological characteristics. Ancillary imaging features such as lobular shape, capsular retraction, segmental biliary dilatation, and vascular encasement favor the diagnosis of mICC. Nevertheless, these radiological findings may also be present in certain benign conditions such as focal confluent fibrosis, sclerosing hemangioma, organizing hepatic abscess, or the pseudosolid form of hydatid disease. In addition, a few malignant lesions including primary liver lymphoma, hemangioendothelioma, solitary hypovascular liver metastases, and atypical forms of hepatocellular carcinoma (HCC), such as scirrhous HCC, infiltrative HCC, and poorly differentiated HCC, may also pose a diagnostic dilemma by simulating mICC in imaging studies. Diffusion-weighted imaging and the use of hepatobiliary contrast agents might be helpful for differential diagnosis in certain cases. The aim of this manuscript is to provide a comprehensive overview of mICC imaging features and to describe useful tips for differential diagnosis with its potential mimickers.

## 1. Introduction

Intrahepatic cholangiocarcinoma (ICC) is the second most common primary malignant liver tumor after hepatocellular carcinoma (HCC) [1]. These tumors arise from the intrahepatic biliary duct epithelium, proximal to the second-order bile ducts [2]. Although the majority of cases occur sporadically, there are certain medical conditions that are considered to be risk factors for ICC development, in particular primary sclerosing cholangitis (PSC), choledochal cyst, intrahepatic lithiasis, Caroli disease, clonorchiasis, and viral hepatitis (especially type C) [3,4].

According to the growth pattern, ICC can be classified into mass-forming, periductal-infiltrating, or intraductal growth types [5]. Among the three different growth patterns, mass-forming cholangiocarcinoma (mICC) is the most common, accounting for 80% of all cases [5]. The second most common type is mixed type consisting of mass-forming and periductal infiltrating growth pattern [5]. Even though mixed type was initially introduced as a distinctive type, it is now grouped together with mass-forming type according to its imaging presentation [5,6]. However, recent studies have shown that gross morphological classification is insufficient in explaining the atypical presentations of ICC [7]. In order to provide better understanding of varying imaging appearances of mICC and its correlation with clinicopathological features, Kim et al. have proposed new dichotomous imaging classification introducing “parenchymal” and “ductal” types of mICC [8]. This is in accordance with the new histological classification that divides ICC into small duct and large duct types [9]. With regard to new imaging classification, parenchymal type originates from small bile ducts or canals of Hering and presents as a mass-forming lesion without gross involvement or bile duct dilatation [8]. On the other hand, ductal type develops from mature cholangiocytes of the large bile ducts and presents usually as mixed mass-forming and periductal infiltrating lesion causing biliary dilatation [8,10]. In addition, it has been shown that the ductal type tends to show hypovascularity while the parenchymal type frequently displays hypervascularity on imaging studies [11,12]. Furthermore, Hayashi et al. have shown that large bile duct type was more commonly associated with poor differentiation due to the rich fibrous stroma in contrast to the small duct type, which had better postsurgical outcomes [12,13]. Therefore, recognition of this different imaging appearances of mICC provides additional clinical information regarding the prognosis and clinical outcome, which may influence treatment decisions in certain cases [8].

Taking into account the variability of mICC presentation, the precise preoperative diagnosis may sometimes represent a real diagnostic challenge even for experienced radiologists. Additionally, atypical appearance of different benign and some malignant lesions may resemble mICC on imaging. Therefore, the aim of this study is to provide a comprehensive overview of typical and atypical imaging features of mICC, as well as to describe the wide spectrum of benign and malignant lesions that can simulate its appearance on magnetic resonance imaging (MRI). Moreover, useful tips for the differentiation of mICC from its potential mimickers are highlighted.

## 2. Imaging Findings of Mass-Forming ICC

The imaging appearance of mICC depends on its pathohistological composition and is determined by the amount of fibrous stroma, viable tumor cells, intralesional mucin, and necrosis [14].

### 2.1. Typical Imaging Features of mICC

**Figure 1:**
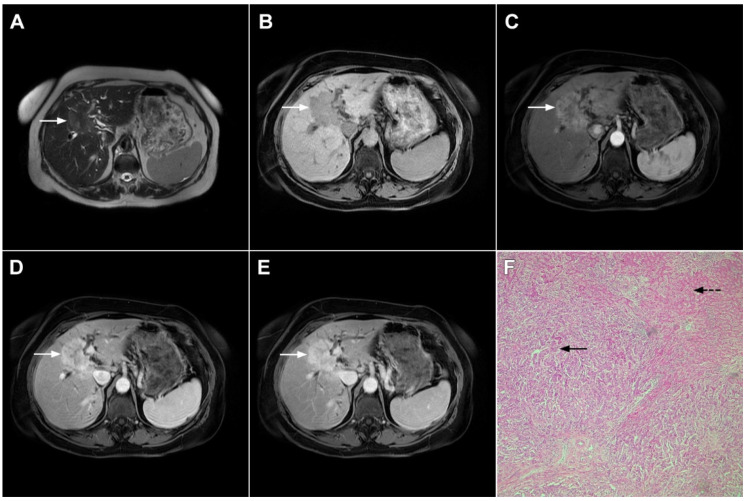
Typical intrahepatic mass-forming cholangiocarcinoma in 68-year-old woman. On axial T2-weighted image a lobular heterogeneously hyperintense tumor (*arrow*) is seen, located centrally in the liver segment IVB (**A**). The lesion (*arrow*) is hypointense in a plain T1-weighted image (**B**) with irregular ring enhancements in the arterial phase (**C**) and progressive enhancement in the portalvenous (**D**) and delayed phase (**E**). Note the perilesional biliary dilatation. Hematoxylin and eosin (H&E) staining (**F**) showed cholangiocarcinoma (*arrow*) and normal liver parenchyma next to the tumor (*dashed arrow*); original magnification ×40.

Mass-forming ICC typically presents as a large, lobulated, irregularly shaped lesion with well-defined borders [14]. On MRI, the tumor is usually hypointense on T1-weighted images, while the appearance on T2-weighted images varies from hypointensity in highly fibrotic lesions to hyperintensity in necrotic or mucin-rich tumors [15]. Although central T2-weighted hypointensity is considered a characteristic MRI feature of mICC, it can also be seen in colorectal metastasis due to intralesional coagulative necrosis [14]. Nevertheless, in most of the cases mICC presents as a heterogeneous lesion on T2-weighted images containing both areas of hyperintensity and areas of hypointensity [14,15]. The characteristic enhancement pattern using conventional gadolinium-based extracellular agents consists of an irregular ring enhancement on the arterial phase followed by progressive central enhancement in the portal venous and delayed phases (Figure 1) [14,15]. This postcontrast behavior could be explained by a rim of viable cells at the periphery of the tumor and rich edematous internal fibrous stroma [15].

**Figure 2:**
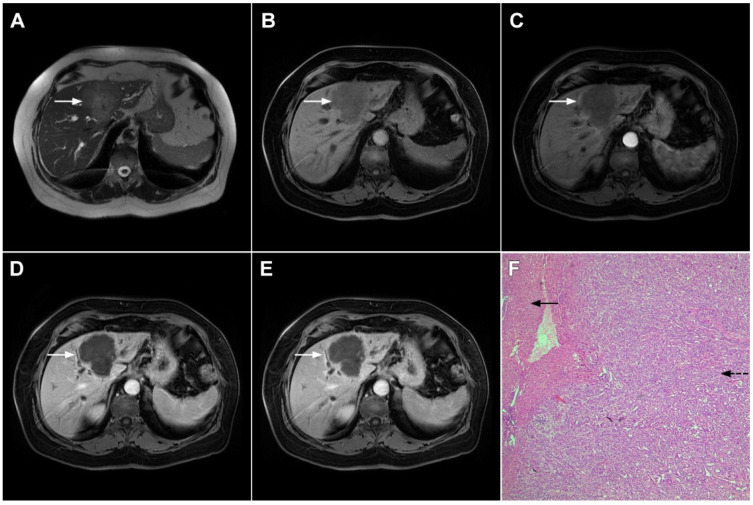
Mass-forming intrahepatic cholangiocarcinoma in 72-year-old man. Irregular heterogeneously hyperintense lesion (*arrow*) on T2-weighted image (**A**) located in liver segments IVB and III with peripheral biliary dilatation is shown. On a plain T1-weighted image (**B**) the lesion (*arrow*) is hypointense with only discrete ring enhancement in the arterial phase (**C**) but without detectable enhancements in the portal venous (**D**) and delayed phases (**E**). Hematoxylin and eosin (H&E) staining (**F**) showed poorly differentiated cholangiocarcinoma (*dashed arrow*); normal liver parenchyma is also shown (*arrow*); original magnification ×40.

However, in highly fibrotic lesions or in lesions with internal necrosis, central parts may remain non-opacified even on delayed images (Figure 2) [14].

**Figure 3:**
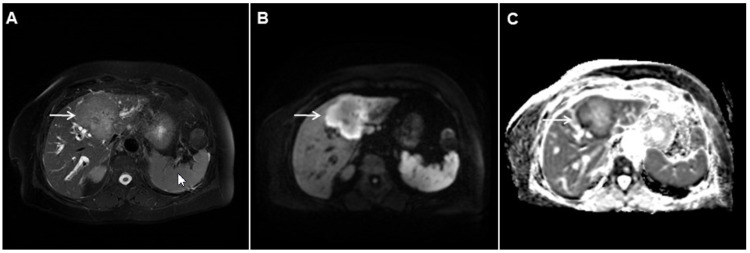
Mass-forming intrahepatic cholangiocarcinoma in the left liver lobe of a 76-year-old man. Axial T2-weighted FS image shows lobulated hetrogeneously hyperintense hepatic tumor (*arrow*) with perilesional biliary dilatation (**A**). Axial diffusion-weighted image (b = 800 s/mm^2^) shows target-like appearance (*arrow*) of the lesion that consists of a central darker area and a peripheral hyperintense area (**B**). Corresponding ADC map is shown on (**C**).

Target sign, consisting of diffusion restriction at the periphery of the lesion and low signal intensity in its central parts on high-*b*-value diffusion weighted imaging (DWI), could be considered to be a pathognomonic finding in mICC [16,17]. It can be attributed to the loose central fibrotic stroma with accompanying edema that is responsible for low signal intensity in DWI, while the periphery of the lesion is composed of densely packed viable tumor cells that cause diffusion restriction and dark rings on the apparent diffusion coefficient (ADC) map (Figure 3) [16].

**Figure 4:**
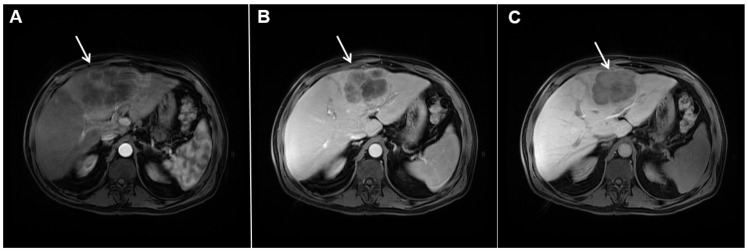
Mass-forming intrahepatic cholangiocarcinoma in a 68-year-old woman. Axial T1-weighted image after gadoxetic acid administration obtained in arterial phase (**A**) shows peripherally enhancing lesion (*arrow*). Portal venous phase in the same patient (**B**) shows progressive centripetal enhancement of the lesion (*arrow*) with cloud-like appearance in the hepatobiliary phase (**C**) consisting of an area of central enhancement and a thin, peripheral, hypointense rim.

If hepatocyte-selective contrast media are used, mICC presents typically as hypointense lesions on the hepatobiliary phase due to the lack of functional hepatocytes with a sharp margin between the tumor and the background liver parenchyma [7]. Thus, tumor size as well as the presence of perilesional satellite nodules, could be more precisely evaluated in the hepatobiliary phase in comparison to the MRI with conventional extracellular contrast [18,19]. Additionally, the “cloud sign”, seen as a relatively high cloud-like signal intensity in the central part of the lesion surrounded by a hypointense peripheral rim, is considered characteristic of mICC (Figure 4) [19]. The appearance of mICC in the hepatobiliary phase may be used as a prognostic factor since it correlates well with the content of intralesional fibrous stroma [20]. Namely, if cloud sign is seen it indicates a large amount of fibrous stroma in the central parts of the tumor, which is frequently associated with poor prognosis [20]. Additional imaging features, which are shown to be prognostic factors and can be assessed in the hepatobiliary phase, include capsule penetration and hepatic vein obstruction, as was demonstrated in the study by Kim S et al. [21].

**Figure 5:**
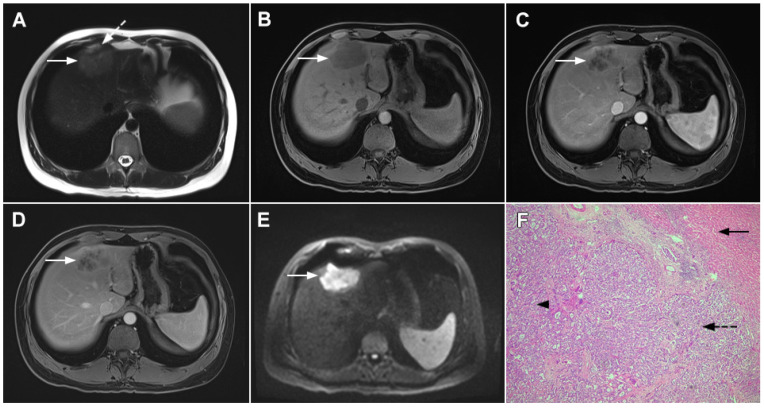
Parenchymal mass-forming cholangiocarcinoma in a 36-year-old man. The lobular slightly hyperintense lesion (*arrow*) is seen in the liver segment IVA in a T2-weighted image (**A**) with subtle capsular retraction (*dashed arrow*). On a plain T1-weighted image (**B**), the tumor (*arrow*) is hypointense with irregular discrete peripheral and central enhancements in the arterial phase (**C**), mild progressive enhancement in the portal venous phase (**D**), and high signal intensity in DWI (**E**). Hematoxylin and eosin (H&E) staining (**F**) showed cholangiocarcinoma (*arrowhead*) with poorly differentiated components (*dashed arrow*). Normal liver parenchyma is also shown (*arrow*); original magnification ×40.

Ancillary MRI findings that are frequently seen in mICC include peripheral biliary dilatation, capsular retraction, vascular encasement, lobar atrophy, satellite nodules, and lymphadenopathy [15,16]. Nevertheless, it should be kept in mind that in the parenchymal type of mICC, due to its origin from the small bile ducts, ancillary features such as biliary dilatation, vascular encasement, and lobar atrophy may be absent (Figure 5). In such cases, the presence of typical postcontrast behavior and capsular retraction indicate mICC. On the other hand, some degree of obstruction and peripheral bile duct dilatation is always seen in the ductal type of mICC [15].

Although capsular retraction is considered to be a characteristic finding for mICC [14,15], it can also be observed in many other lesions with abundant fibrosis and desmoplasia, such as metastatic colon carcinoma, scirrhous hepatocellular carcinoma, and hemangioendothelioma [22]. Therefore, this sign must be evaluated only in combination with other imaging findings. Due to its infiltrative growth, mICC may lead to vascular encasement, resulting in lobar or segmental parenchymal atrophy [14,15]. In contrast to HCC, mICC rarely leads to tumor thrombus formation [23]. Satellite lesions around the main tumor are frequent findings probably due to the invasion of adjacent small portal vein branches [19]. The formation of satellite nodules, and their subsequent fusion with the main tumor, results in the lobular shape of the lesion, which is shown to be a characteristic feature of mICC [19]. Lymphadenopathy in porta hepatis, and hepatoduodenal ligament can be seen in up to 73% of mICC cases but is a nonspecific finding [15].

### 2.2. Atypical Imaging Features of mICC

**Figure 6:**
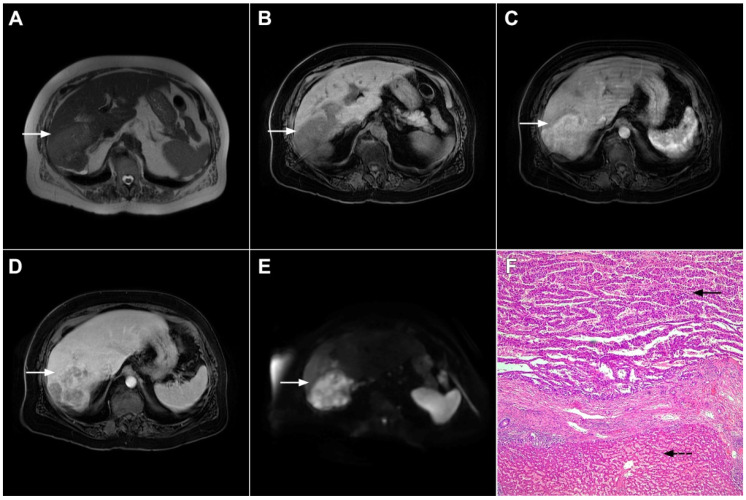
Hypervascular mass-forming cholangiocarcinoma in a 63-year-old woman. The axial T2-weighted image (**A**) shows a moderately hyperintense tumor (*arrow*) located in liver segments VI and VII with a subtle medial capsular retraction. The lesion (*arrow*) is hypointense on the plain T1-weighted image (**B**), hypervascular in the arterial phase (**C**) with washout on the portal venous phase (**D**). The tumor (*arrow*) is hyperintense on DWI (**E**). Hematoxylin and eosin (H&E) staining (**F**) showed well-differentiated cholangiocarcinoma (*arrow*) surrounded by normal liver parenchyma (*dashed arrow*); original magnification ×40.

Besides the typical imaging presentation of mICC, atypical appearance can also be observed in imaging studies [24]. Hypervascular mICCs are usually small lesions and this vascular behavior might be explained by less intralesional fibrosis and abundant vascular stroma [25]. The incidence of hypervascular mICC ranges from 12.5% up to 47% in previous reports [18,25]. Since hypervascular mICCs are frequently seen in cirrhotic livers, the differential diagnosis with HCC may be very difficult. In this regard, the absence of washout and the presence of progressive enhancement together with the lack of fibrous pseudocapsule favor the diagnosis of mICC over HCC [25]. However, if hypervascular mICC shows washout in the portal venous phase, preoperative differential diagnosis with HCC is hardly possible (Figure 6). In such cases, additional findings, such as cloud appearance in the hepatobiliary phase, multiplicity around the main tumor, or intrahepatic metastasis, capsule retraction and tumor markers may be helpful for differentiating between these tumors [18,19]. Hypervascular mICC differs from typical hypovascular mICC not only in terms of vascularity but also in patient outcome, as it has been shown that the former has much better prognosis [12]. Therefore, the assessment of tumor vascularity on preoperative imaging could represent an important marker for predicting malignant characteristics in mICC [26].

**Figure 7:**
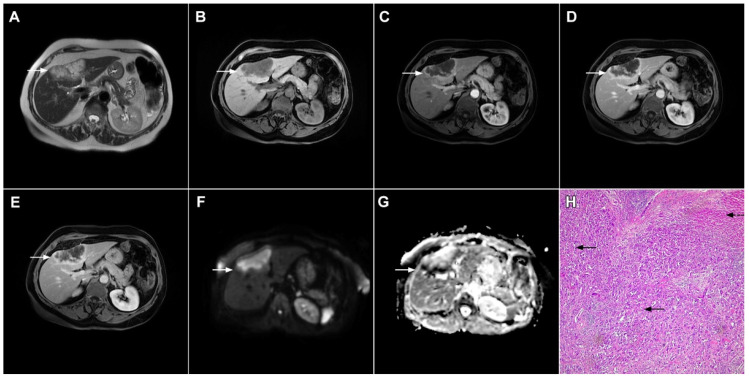
Mucin-rich mass-forming cholangiocarcinoma in a 78-year-old woman. The axial T2-weighted image (**A**) shows the lobulated hyperintense lesion (*arrow*) located in the subcapsular region of liver segment IVB, which is associated with capsular retraction. On the plain T1-weighted image (**B**) the lesion (*arrow*) is hypointense. In the arterial phase (**C**), ring enhancement can be seen with slight “ragged” central enhancement in the portal venous (**D**) and delayed phase (**E**). On DWI, diffusion restriction is noted on the periphery of the lesion (*arrow*) while no restriction is seen in the central part of the tumor (**F**). Corresponding ADC map showing targetoid appearance of the lesion is shown on (**G**). Hematoxylin and eosin (H&E) staining (**H**) showed cholangiocarcinoma (*arrows*) adjacent to normal liver parenchyma (*dashed arrow*); original magnification ×40.

Mucinous cholangiocarcinoma is a rare variant of mICC characterized by rich mucin production [24,27]. According to previous studies, mucinous mICC originate from mucin-producing cholangiocytes located in large bile ducts [28]. Imaging findings in mucinous mICC reflect characteristic histopathological features of the lesion with cancer cell nests suspended in a large mucinous lake [29]. Therefore, these lesions display strong T2-weighted hyperintensity [27]. Moreover, as cancer cells in the center of the lesion are scarce, these tumors show only slight progressive enhancement of intralesional septa and cellular nests in postcontrast studies (Figure 7) [27]. Due to its very high signal intensity on T2-weighted images and centripetal pattern of enhancement, mucinous mICC may be misdiagnosed as hemangiomas [29]. Concerning similarities in the postcontrast behavior of mucinous mICC and hemangiomas, it should be kept in mind that mICC displays continuous ragged rim enhancements in contrast to the discontinuous, stronger peripheral and centripetal enhancement following the blood pool seen in hemangiomas [24,29].

## 3. Mimickers of Mass-Forming ICC on MRI

### 3.1. Benign Lesions

#### 3.1.1. Focal Confluent Fibrosis

Focal confluent fibrosis develops in chronic liver diseases as a result of extensive parenchymal fibrosis [30]. It is usually seen in patients with alcoholic cirrhosis and long-standing primary sclerosing cholangitis [31].

**Figure 8:**
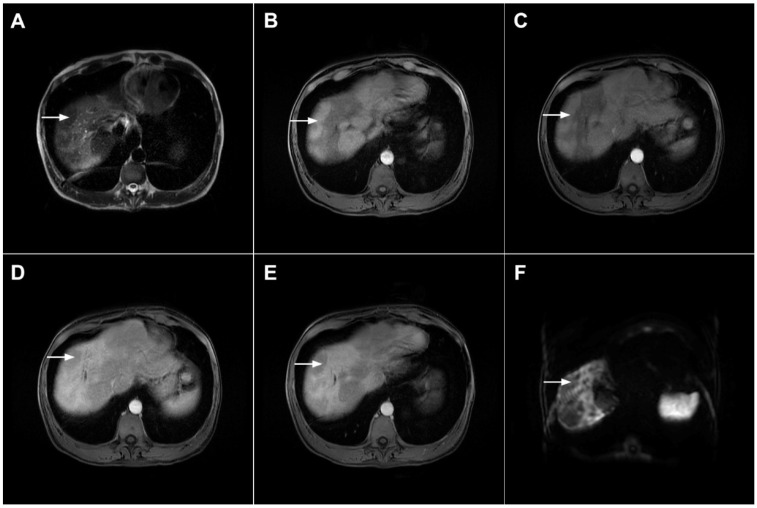
Focal confluent fibrosis in a 42-year-old man with long-standing primary sclerosing cholangitis. The T2-weighted image (**A**) shows a band like a slightly hyperintense lesion (*arrow*) at the dome of the liver. On the plain T1-weighted image (**B**) the lesion (*arrow*) is hypointense without arterial vascularity (**C**) while homogeneous progressive enhancement is seen in the portal venous (**D**) and delayed phases (**E**). The lesion (*arrow*) shows high signal intensity in DWI (**F**).

MRI features of focal confluent fibrosis may mimic mICC [30]. Since both mICC and confluent fibrosis develop in the same group of patients, it is very important to draw a distinction between these two entities. Similarly to mICC, focal confluent fibrosis shows mild to moderate hyperintensity on T2-weighted images and capsular retraction [30]. However, while mICC presents as a lobulated lesion associated with capsular retraction, focal confluent fibrosis is typically seen as a wedge-shaped lesion radiating from the porta hepatis with straight or concave borders and the base in the subcapsular region [15,30]. Focal confluent fibrosis commonly involves segments IV, VII, or VIII corresponding to the drainage territory of the middle hepatic vein [32,33]. Dilatation of proximal intrahepatic biliary ducts is frequently seen in mICC, whereas it is not observed in focal confluent fibrosis [15]. Focal confluent fibrosis lacks arterial vascularity, with homogeneous mild enhancement during the portal venous phase and strong enhancement in the delayed phase (Figure 8) [34]. Progressive enhancement seen in focal confluent fibrosis, attributed to the accumulation of contrast agents in the extracellular compartment, should not be confused with postcontrast behavior of mICC, which is irregular and heterogeneous [15,30]. Occasionally mild arterial phase enhancement may be present in focal confluent fibrosis, which can be explained by immature fibrosis and inflammation occurring in the early phase of development [30]. In such cases, other imaging features such as the shape of the lesion, associated biliary dilatation, satellite nodules, and lymphadenopathy must be considered. Concerning relationship with vascular structures, it should be noted that trapped and crowded vessels may be seen within confluent fibrosis mimicking vascular encasement, which is frequently present in mICC [30]. The use of hepatobiliary contrast agents usually does not provide additional information to distinguish between mICC and focal confluent fibrosis since both lesions are hypointense in the hepatobiliary phase [35]. Nevertheless, the visualization of recently described cloud signs favors the diagnosis of mICC [19]. In addition, the target sign on high *b*-value DWI is frequently observed in mICC, whereas focal confluent fibrosis displays only slight homogeneous diffusion restriction, but with ADC the values slightly higher than those of mICC [16].

#### 3.1.2. Sclerosing Hemangioma

Liver hemangiomas are the most common benign liver tumors [36]. If the characteristic pattern of discontinuous, peripheral, globular enhancement with subsequent central fill-in and sustained enhancement in delayed phases is present, the diagnosis of hemangioma is straightforward [37]. In addition, high signal intensity in T2-weighted images representing a “light bulb” sign, attributed to slow blood flow in the vascular spaces, supports the diagnosis of hemangiomas [37]. Moreover, on DWI most hemangiomas display a high signal intensity that is not caused by restricted diffusion but rather by the T2 shine-through effect [38].

**Figure 9:**
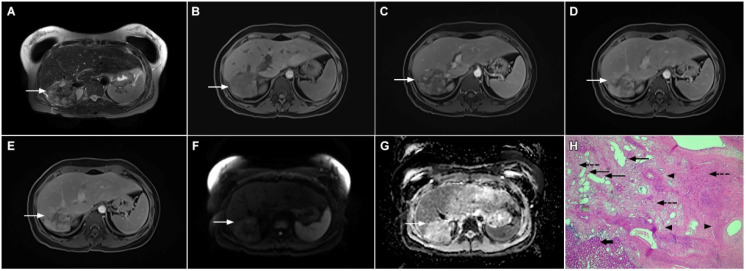
Sclerosing hemangioma in a 39-year-old woman. On T2-weighted, fat-suppressed image (**A**) a heterogeneous lesion (*arrow*) is located in liver segment VII and the upper part of segment VI is shown. Note both hyperintense and hypointese intermingled areas inside the lesion. On the plain T1-weighted image (**B**), the lesion is hypointense (*arrow*) with small, patchy areas of intense enhancement in the arterial phase (**C**), which enlarge in the portal venous (**D**) and delayed phases (**E**). The lesion (*arrow*) does not show diffusion restriction (**F**) and has high signal intensity in the corresponding ADC map (**G**). Hematoxylin and eosin (H&E) staining (**H**) showed sclerosing hemangioma containing multiple small blood vessels with thin walls (*thin arrows*), blood vessels with thick walls (*arrowheads*), and areas of fibrosis and sclerosis (*dashed arrows*). Liver parenchyma is also shown (*thick arrow*); original magnification ×40.

In rare cases hemangiomas may present with atypical imaging features mimicking malignant hepatic lesions [39]. Minor hemorrhage and thrombosis within a hemangioma may initiate fibrotic process leading to the development of sclerosing hemangioma [40]. If vascular stroma is completely replaced by fibrosis, these hemangiomas are called sclerosed hemangiomas [40]. Degenerative changes impact the imaging presentation of hemangioma, making differential diagnosis with malignant lesions challenging [39,41]. In contrast to the characteristic high signal intensity in T2-weighted images, sclerosing hemangioma may display heterogeneous, slight T2-weighted hyperintensity or even hypointensity [39,41]. Depending on the amount of fibrosis in sclerosing hemangiomas, different types of enhancement may occur, including a lack of early nodular peripheral opacification, thick ring arterial phase enhancement, or persistent mild peripheral enhancement [42]. Furthermore, in the study by Shin et al. centripetal patchy enhancement with a partial unenhanced area on CT and MRI was considered highly suggestive of sclerosing hemangioma [42]. Nevertheless, if progressive central opacification is seen in conjunction with capsular retraction, differential diagnosis with mICC might be very difficult [43]. To overcome potential pitfalls, the presence of nodular enhancement in parts of the lesion’s geographic margins decrease in size at follow-up, the presence of transient hepatic attenuation differences, and the loss of previously seen regions of enhancement favor the diagnosis of sclerosing hemangioma [44]. Moreover, the presence of high signal intensity areas in T2-weighted images inside the lesion that correspond to the foci of enhancement in the arterial phase with sustained and progressive opacification in the delayed phases, mixed with T2-weighted hypointense areas, should raise the possibility of sclerosing hemangioma (Figure 9) [39]. Nevertheless, in cases where differential diagnosis with mICC is not possible, a percutaneous biopsy is advised.

#### 3.1.3. Inflammatory Pseudotumor

Inflammatory pseudotumor (IPT) represents a rare focal liver lesion composed of variable amount of fibrosis, foamy histiocytes, plasma cells, lymphocytes, and macrophages without cellular atypia [45,46]. The etiology is still unclear, although infection, trauma, immunologic reaction, or primary biliary cholangitis have been proposed as predisposing factors [45]. The occurrence of hepatic IPTs has been reported in all age groups, but it is most commonly seen in children and young adults [45,46].

**Figure 10:**
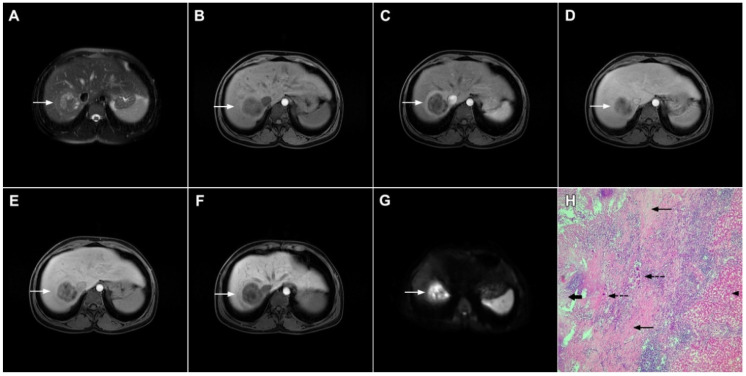
Inflammatory pseudotumor in a 38-year-old woman. On T2-weighted fat-suppressed image (**A**), a round lesion (*arrow*) is seen in the central part of liver segment VII. An isointense rind surrounding the heterogeneous central part of the lesion can be noted. The lesion (*arrow*) is hypointense in a plain T1-weighted image (**B**) with enhanced rims in the arterial phase (**C**). In the portal–venous (**D**) and delayed phases (**E**)**,** slight progressive central enhancement is seen without washout on the periphery of the lesion. In the hepatobiliary phase, the lesion (*arrow*) is hypointense centrally while the peripheral rim is isointense with the surrounding liver parenchyma (**F**). The lesion (*arrow*) shows high signal intensity on DWI (**G**). Hematoxylin and eosin (H&E) staining (**H**) showed inflammatory pseudotumor with foreign body giant cells and Langerhans cells (*dashed arrows*); foci of necrosis (*thick arrow*) surrounded by fibro-inflammatory capsule (*thin arrows*). Normal liver parenchyma is also seen (*arrowhead*); original magnification ×40 (**H**).

The imaging characteristics of IPT are diverse, depending on the proportion of inflammatory cells and fibrous stroma within the lesion [47]. On MRI, IPTs usually present as solitary, well-defined, heterogeneously T2-weighted hyperintense, T1-weighted hypointense lesions with restricted diffusion [48]. The postcontrast behavior pattern is highly variable, ranging from peripheral enhancement in the arterial phase to progressive central enhancement up to non-enhancement or even hyperenhancement in the arterial phase with washout in the portal–venous phase (Figure 10) [47,48].

**Figure 11:**
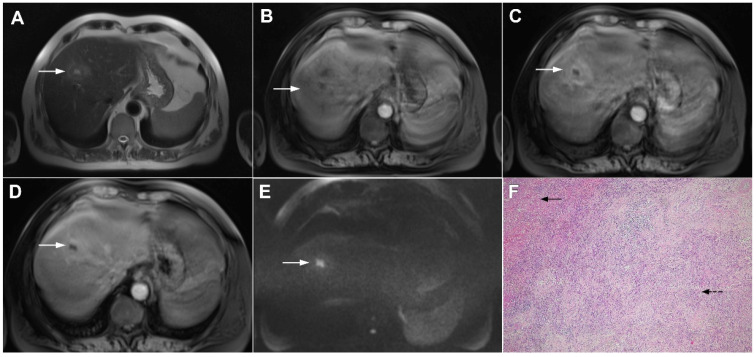
Inflammatory pseudotumor in a 68-year-old man. In the T2-weighted image (**A**), a round lesion (*arrow*) is seen in the central part of the liver segment VIII. A central necrotic area can be seen surrounded by an irregular rim, which is isointense in the plain T1-weighted image (**B**), well-vascularized in the arterial phase (**C**), and has a persistent enhancement in the portal–venous phase (**D**). In the DWI, only the central necrotic part (*arrow*) shows high signal intensity, while the peripheral rim is isointense with the surrounding liver parenchyma (**E**). Hematoxylin and eosin (H&E) staining (**F**) showed inflammatory pseudotumors rich with myofibroblasts and inflammatory cells (*dashed arrow*). The normal liver parenchyma is also marked (*arrow*); original magnification ×40 (**F**).

A thick rind composed of fibrosis and inflammatory cells intermingled with hepatocytes that typically shows delayed postcontrast enhancement is frequently seen in IPTs (Figure 11).

Due to the atypical imaging presentation, IPT is usually misdiagnosed as other benign or malignant liver lesions such as mICC, HCC, or a hepatic abscess [49,50]. The differentiation from mICC might be challenging if IPT demonstrates an enhancement pattern similar to mICC [51]. In this reagrd, Chang et al. have pointed out the differences regarding the targetoid appearance of IPT in the early phase of dynamic study, whereas mICC demonstrates a targetoid appearance in the hepatobiliary phase in DWI [51]. Additionally, in 75.0% of IPT cases, central iso- and peripheral hyperintensity on T2-weighted images was found, while 84.4% of ICCs showed layered hyperintensity with either brighter or darker areas in the center [51]. Furthermore, if peripheral biliary dilatation, satellite lesions, and capsular retraction are present, the diagnosis of mICC should be favored over IPT [50,51]. Nevertheless, in addition to imaging presentation it is equal to consider epidemiological and clinical data, since younger age and the presence of inflammatory syndrome raise the suspicion of IPT [45,46,47]. Taking into account that some IPT may regress spontaneously or after conservative treatment, preoperative differentiation between IPT and mICC is very important [49,50]. Therefore, in all doubtful cases a percutaneous liver biopsy should be performed before surgical resection.

#### 3.1.4. Pyogenic Liver Abscess

Pyogenic liver abscess is a common disease, with Gram-negative bacteria being the most common causative factor [52]. Characteristic imaging findings include a central fluid-like pus collection with a multilayered rim known as a “double target sign” [52,53]. Another typical imaging feature frequently seen in liver abscess is a “cluster sign”, which represents the confluence of multiple small locules in a localized area to form a solitary, large abscess cavity [54].

**Figure 12:**
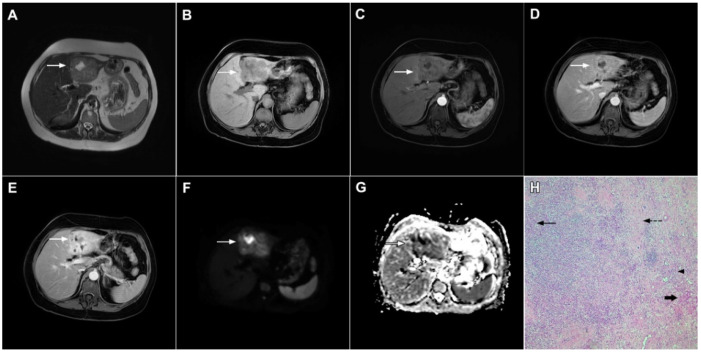
Solid organizing liver abscess in a 44-year-old woman. A T2-weighted slightly hyperintense lesion (*arrow*) is seen in liver segment III (**A**). Note the intralesional, eccentrically located hyperintense area representing necrosis. The lesion (*arrow*) is hypointense in the plain T1-weighted image (**B**) with a subtle enhancement in the arterial phase (**C**) and progressive opacification in the portal venous (**D**) and delayed phases (**E**). In the DWI, the lesion (*arrow*) shows mild diffusion restriction except the small area representing necrosis, which displays high signal intensity (**F**). The corresponding ADC map is shown in (**G**). Hematoxylin and eosin (H&E) staining (**H**) showed a solid organizing liver abscess with purulent absceding inflammation (*thin arrow*), a hyalinized acellular fibrous capsule (*dashed arrow*), and multiplied biliary ductules (*arrowhead*). Normal liver parenchyma is also shown (*thick arrow*); original magnification ×40.

However, the imaging findings of hepatic abscesses may vary, with a solid organizing form being a rare manifestation that might occur in the chronic stage of the disease. Due to the advanced organizing process or granulomatous features, the lesion appears mostly solid without abundant pus collection [55]. As for its unspecific imaging features, solid organizing abscess may simulate malignant lesions, in particular mICC [55,56]. Namely, in the arterial phase both lesions might show peripheral ring enhancement. Nevertheless, in the portal–venous and delayed phases, mICC typically shows peripheral washout with central progressive enhancement [57], while no washout is seen in a solid organizing hepatic abscess [57]. Moreover, reticular progressive internal opacification is frequently observed in the solid form of hepatic abscesses (Figure 12) [55].

**Figure 13:**
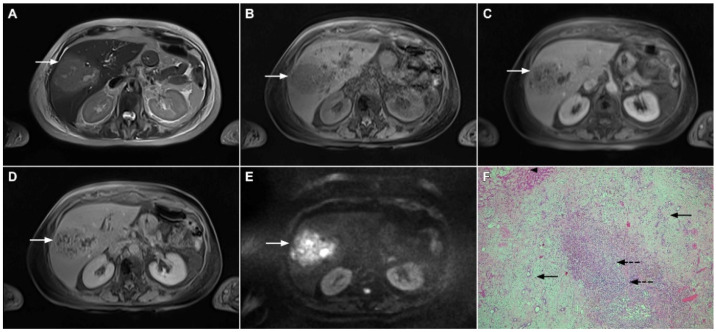
Focal chronic liver abscess in a 77-year-old woman. In the T2-weighted image (**A**), a mildly hypertentense lesion (*arrow*) is seen in liver segment V. Note also a few intralesional foci of high signal intensity representing necrosis. The lesion (*arrow*) is hypointense in the plain T1-weighted image (**B**) with subtle reticular internal opacification in the late arterial phase (**C**) and progressive enhancement in the portal venous phase (**D**). In the DWI (**E**), the lesion (*arrow*) shows high signal intensity with internal spots of very high signal intensity corresponding to necrosis. Hematoxylin and eosin (H&E) staining (**F**) showed a solid organizing liver abscess with foci of necrosis (*dashed arrows*) and reactive ductal hyperplasia (*arrows*) surrounded by normal liver parenchyma (*arrowhead*); original magnification ×40.

In addition, the presence of tiny fluid-like spaces in the center of the lesion corresponding to small necrotic portions might be a helpful clue for the differential diagnosis as they are seen only in solid organizing abscesses (Figure 13) [55]. Other imaging findings, such as areas of perilesional edema and transient early segmental enhancement, favor the diagnosis of the inflammatory process over mICC [53]. On the contrary, capsular retraction, biliary duct dilatation upstream to the mass, lobar or segmental atrophy and vascular encasement are suggestive of mICC.

Another potential diagnostic pitfall is related to the presence of extensive intralesional necrosis in mICC, which may simulate hepatic abscesses [58]. Although differential diagnosis might be very difficult, especially if the patient presents with inflammatory symptoms, the analysis of the wall enhancement and internal signal intensity of the lesion should be used to make the correct diagnosis. Namely, the postcontrast enhancement of the wall of necrotic mICC consists of irregular rim enhancement in the arterial phase with washout in delayed phases, in contrast to the typical “target” appearance of liver abscess [58]. Moreover, the internal surface of the wall of necrotic mICC is nodular and irregular, while the inner wall of the abscess is mostly regular [58]. In addition, the presence of multiple dot or patchy central and wall nodules enhancement suggests the diagnosis of mICC, while in abscess no internal enhancement is noted. Extrahepatic findings such as multiple enlarged lymph nodes in the hepatic hilar are, and the retroperitoneal space further indicate the diagnosis of malignancy [59]. Concerning the differential diagnosis between these two entities, clinical information such as non-resolving suspected liver abscess, especially in the elderly population, should raise concerns of necrotic tumors [56]. Given that liver abscess can regress completely with appropriate conservative treatment, the differentiation between these entities is very important to avoid unnecessary surgery or a delay in the diagnosis of mICC.

#### 3.1.5. Liver Echinococcosis

**Figure 14:**
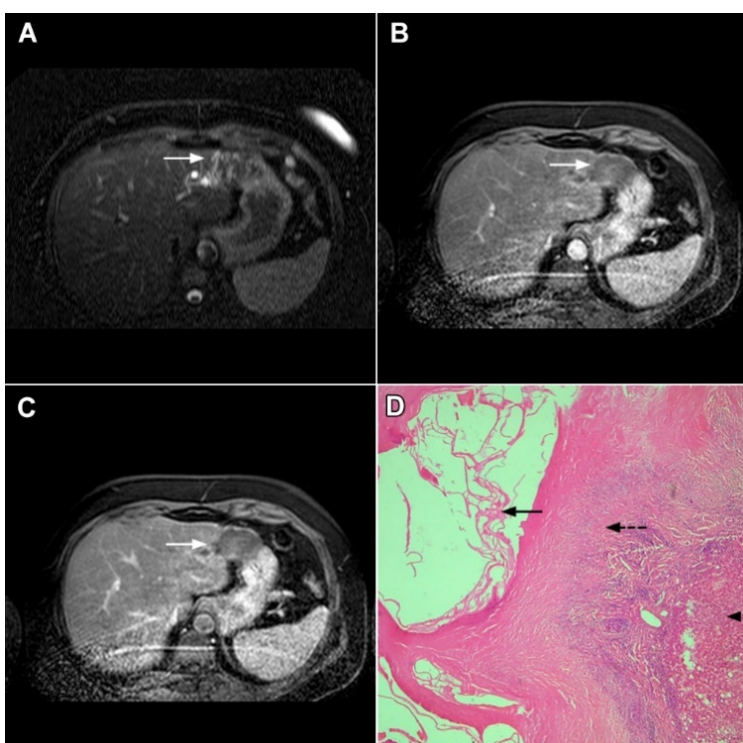
Solid-appearing liver echinococcosis in a 54-year-old woman. An axial T2-weighted FS image (**A**) shows a heterogeneous liver lesion (*arrow*) in liver segment II with internal hypointense areas. A slight biliary dilatation adjacent to the lesion can also be seen. The lesion (*arrow*) is hypointense in the arterial phase (**B**) and remains hypointense in the portal venous phase (**C**), simulating a hypovascular liver tumor. Hematoxylin and eosin (H&E) staining (**D**) showed an echinococcal cyst with germinative membranes (*arrow*) and the thick hyalinized wall of the cyst (*dashed arrow*) surrounded by normal liver parenchyma (*arrowhead*); original magnification ×40.

Hydatid liver disease results from incidental infection with Echinococcus granulosus, which causes cystic echinococcosis, or Echinococcus multilocularis, which causes alveolar echinococcosis (AE) [60]. If characteristic imaging features are present in the form of a cystic lesion with multiple internal daughter cysts and the absence of postcontrast enhancement, there are no difficulties in establishing the correct diagnosis [61]. However, in cases of inactive infection with solid-appearing pseudotumors making the correct diagnosis might be difficult, and in certain cases mICC may be suspected [62]. Although the absence of postcontrast enhancement is a typical finding in solid-appearing liver echinococcosis, a mild delayed peripheral opacification corresponding to the fibroinflammatory rim may be present [62]. In such cases, the visualization of dystrophic calcifications is helpful to distinguish between hydatid disease and mICC (Figure 14) [62]. Since the detection of calcifications is difficult via MRI examinations, in doubtful cases an additional CT examination should be performed.

**Figure 15:**
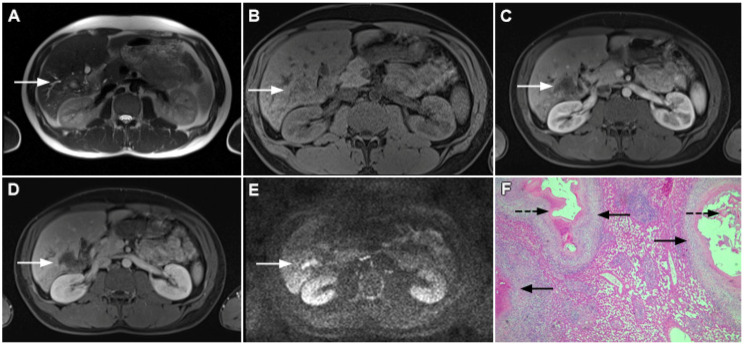
Alveolar echinoccosis in a 31-year-old man. An irregularly shaped lesion (*arrow*) is seen in liver segment VI, presenting as heterogeneously slightly hyperintense in the axial T2-weighted image (**A**) and hypointense in the plain T1-weighted image (**B**). Note the perilesional biliary dilatation. No enhancement is detected in the central part of the lesion (*arrow*), while there is subtle enhancement in the posteromedial part of the lesion in the arterial (**C**) and portal venous phases (**D**). The lesion does not show restricted diffusion (**E**). Hematoxylin and eosin (H&E) staining showed alveolar echinococcosis with multiple multilocular cysts (*arrows*) and hydatid membranes (*dashed arrows*) (**F**); original magnification ×40.

While the diagnosis of a typical hepatic hydatid cyst is in most cases straightforward, the alveolar form of hydatid disease frequently represents a diagnostic challenge [63,64]. According to Kodama et al., AE may present in one of five distinct ways in an MRI scan [65]. Among them, type 2 with multiple small round cysts and a solid component, type 3 with a solid component containing multiple small cysts surrounding a large, irregular pseudocyst, and type 4 presenting as a heterogeneous infiltrative mass with irregular borders might be confused with hepatic malignant tumors, especially mICC [65]. In this regard, the presence of an irregular, infiltrative hypovascular lesion with mild peripheral enhancement in the delayed phase may be easily misinterpreted as mICC (Figure 15) [62,63,66]. Additionally, if large areas of necrosis in hepatic alveolar echinococcosis are present, necrotic mICC may be suspected [64,66]. Furthermore, ancillary imaging features that are considered characteristic of mICC, such as the invasion of the portal vein leading to lobar atrophy, biliary dilatation, and capsular retraction, may also be detected in hepatic AE [67]. Concerning the differential diagnosis between these entities, Mueller et al. found that no or septal enhancement in an MRI scan and matrix calcifications seen in a CT scan are the strongest indicators of AE [66]. Moreover, in the context of rim enhancement, which may be present in AE—leading to confusion with mICC—Wa et al. found that rim enhancement of mICC was more irregular and thicker than the linear rim of AE lesions [63]. To prevent potential misdiagnosis, in all cases when AE is suspected CT should be performed in addition to MRI, as it provides for the detection of typical calcifications and the absence of distinct vasculature inside the mass [61,64,65].

### 3.2. Malignant Lesions

#### 3.2.1. Solitary Hypovascular Liver Metastasis

Solitary hypovascular liver metastases (SHLM) are the most common mimickers of mICC [68]. Although in most cases the primary origin of the tumor is known, in a small subset of patients with a metastatic disease the primary site is unknown or undetectable. The inability to detect the primary tumor could be attributed to favorable metastatic ability rather than local tumor growth [69]. Moreover, patients with known malignant disease could develop mICC independently of their primary tumor. In addition, the differential diagnosis between mICC and SHLM may even be histologically very difficult, since liver metastases from pancreatic and gallbladder cancers have similar cytokeratin expressions to mICC [70]. Considering that the treatment of these two entities is quite different, the distinction between them is of great clinical importance since the only potentially curative treatment for mICC is complete surgical resection, while chemotherapy may be an option for some patients with SHLM [71,72,73].

**Figure 16:**
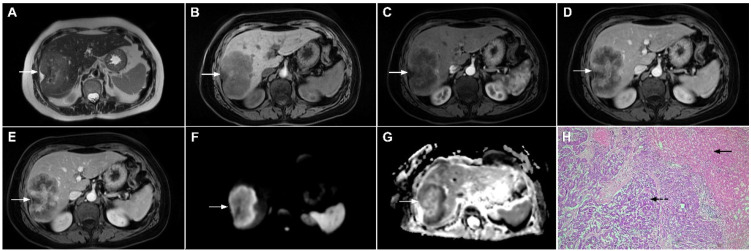
Solitary hypovascular liver metastasis in a 59-year-old woman. A slightly hyperintense lobulated lesion (*arrow*) with capsular retraction is seen in liver segments VI and VII in the T2-weighted image (**A**). The tumor (*arrow*) is hypointense in the plain T1-weighted image (**B**) with a slight peripheral enhancement in the arterial phase (**C**) and a progressive central enhancement in the portal venous (**D**) and delayed phases (**E**). In the DWI (**F**) and corresponding ADC map (**G**), the tumor (*arrow*) shows targetoid appearance. Hematoxylin and eosin (H&E) staining showed well-differentiated adenocarcinoma cells of intestinal type (*dashed arrow*), and normal liver parenchyma adjacent to the metastasis (*arrow*); original magnification ×40 (**H**).

The most common postcontrast enhancement seen in SHLM is ring enhancement in the arterial phase with minimal central enhancement in the delayed phases [68]. This vascular behavior could be explained by the fact that metastatic tumors parasitize the surrounding blood vessels, creating the ring appearance of blood supplying the most vascularized outer parts of the tumor [25]. On the contrary, in mICCs arterial ring enhancement is commonly followed by progressive central enhancement [5,29]. Nevertheless, previous studies have shown that the enhancement characteristics of these two tumors may overlap. Although the central T2-weighted hypointensity and bile duct dilatation proximal of the tumor are considered to be typical for mICC, they can also occur in liver metastases [68]. In such cases, other imaging features, including shape of the lesion, capsular retraction, and portal lymphadenopathy, are suggestive of mICCs [17]. Recent studies have stressed the importance of imaging appearance in DWI for the differential diagnosis. Accordingly, Park et al. found the “target sign” to be a significant predictor of mICC as it was present in 75% of mass-forming ICCs in their study population [16]. Even though all these findings are more frequent in mICC, they can also be observed in SHLM, especially colorectal cancer (Figure 16) [74,75]. In conclusion, considering that both the imaging and pathohistological features of mICC and SHLM significantly overlap, the first step in the diagnosis of mICC should be the exclusion of extrahepatic malignancies, especially colorectal carcinoma.

#### 3.2.2. Atypical Forms of Hepatocellular Carcinoma

##### Scirrhous HCC

**Figure 17:**
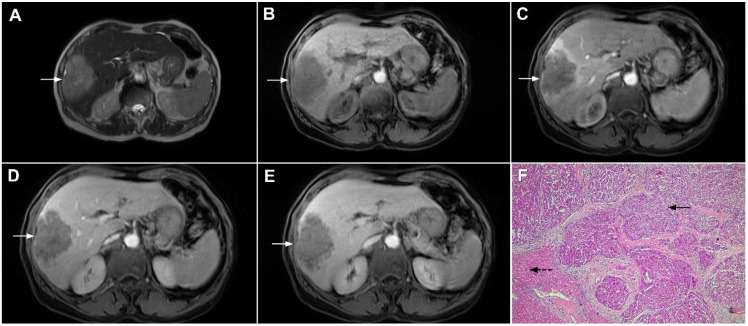
Scirrhous hepatocellular carcinoma in a 68-year-old woman. The axial T2-weighted image (**A**) shows a moderately hyperintense subcapsular-located lesion in liver segments VI and V (*arrow*). Note also the capsular retraction. The tumor (*arrow*) is hypointense in the plain T1-weighted FS image (**B**), with ring enhancement in the arterial phase (**C**) and slight progressive central enhancement in the portal venous (**D**) and delayed phases (**E**). Hematoxylin and eosin (H&E) staining showed hepatocellular carcinoma (*arrow*) and normal liver parenchyma adjacent to the tumor (*dashed arrow*); original magnification ×40 (**F**).

Scirrhous HCC is a rare variant of HCC characterized by rich intralesional fibrotic stroma with incidence of 4.6% among all HCC cases [76]. Scirrhous HCCs are usually seen in MRI scans as lobulated T1-weighted hypointense lesions with a heterogeneous signal intensity on T2-weighted images [77]. With regard to its vascular behavior, Kim et al. showed that the most common enhancement pattern was a peripheral rim-like enhancement in the arterial phase with a progressive central enhancement in the portal–venous and equilibrium phase, which is indistinguishable from mICC (Figure 17) [78]. Moreover, in previous studies washout was seen in only 19% of scirrhous HCC, in comparison to 99.7% of typical HCCs [78]. In addition, similarly to mICC, scirrhous HCC may also cause capsular retraction if it is subcapsular [79]. With regard to the targetoid appearance in the DWI and hepatobiliary phase, both mICC and scirhous HCC can display these imaging features [80]. Therefore, differentiation between scirrhous HCC and mICC represents a real diagnostic challenge, since both lesions have rich fibrous stroma, similar postcontrast behavior, and occur in cirrhotic livers [77]. Concerning different treatment strategies for these tumors, preoperative distinction is very important. In this context, the ancillary feature favoring the diagnosis of mICC include peripheral biliary dilatation, while the presence of a capsule suggests scirrhous HCC [77]. Moreover, the presence of part of the tumor enhancing avidly in the arterial phase favors the diagnosis of scirrhous HCC over mICC [80]. Additionally, Choi SY et al. have shown that mICC more frequently showed T2-weighted central brightness due to the presence of internal necrosis or mucin accumulation [79]. Nevertheless, if the lesion with imaging features of both scirrhous HCC and mICC is seen in the setting of chronic liver disease, according to the Liver Imaging Reporting and Data System (LI-RADS) it is characterized as LI-RADS M and further biopsy is required [81].

##### Poorly Differentiated HCC

**Figure 18:**
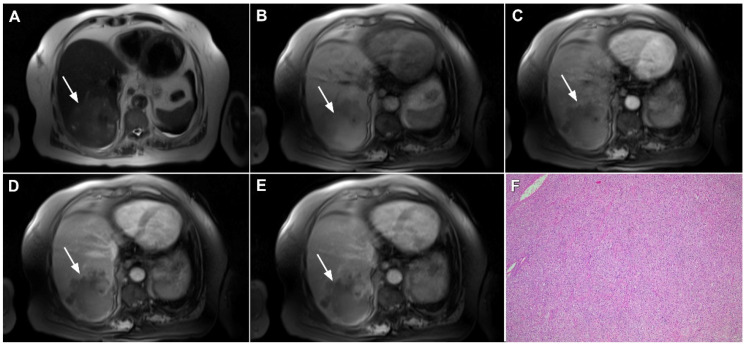
Poorly differentiated hepatocellular carcinoma in a 69-year-old man. The axial T2-weighted image (**A**) shows a moderately hyperintense lesion (*arrow*) in liver segment VII. On plain T1-weighted image (**B**), the tumor (*arrow*) shows central hyperintensity and peripheral hypointensity, with only a slight ring enhancement in the arterial phase (**C**). The tumor (*arrow*) remains centrally hypointense in the portal venous (**D**) and delayed phases (**E**) with irregular nodular peripheral enhancement. Hematoxylin and eosin (H&E) staining showed poorly differentiated HCC; original magnification ×40 (**F**).

The absence of a typical HCC vascular enhancement pattern may be observed in poorly differentiated, large, and sarcomatous HCC [81]. Namely, in sarcomatous HCC neovascularization is not sufficient to supply the rapidly growing sarcomatous component, leading to central ischemia. Similarly, in poorly differentiated HCCs the presence of central no enhancing areas is a frequent finding due to a decrease in the arterial blood supply [82]. As a consequence of internal structural changes, these atypical forms of HCC may show only slight peripheral enhancement, presenting as hypovascular lesions mimicking mICC (Figure 18) [83,84]. In such cases, other imaging features such as capsule and T2-weighted hyperintense foci are found to be significant predictors of atypical HCCs in comparison to mICC [84]. These tiny T2-weighted hyperintense foci can be explained by scattered haemorrhagic foci [84]. In general, internal hemorrhage is considered to be a pathognomonic feature of HCC and was incorporated into LI-RADS as an ancillary feature favoring HCC [85]. Additional findings helpful for discrimination between mICC and poorly differentiated HCC include a lobulated shape, indistinct margin, peripheral rim enhancement in the arterial phase, and the presence of biliary dilatation, which suggest the diagnosis of mICC [82]. Conversely, a round shape, partially indistinct margin, heterogeneous enhancement in the arterial phase, washout pattern, and the presence of tortuous tumoral vessels favor the diagnosis of poorly differentiated HCC [82]. Another finding suggestive of HCC is the presence of intralesional fat, which is never observed in mICC [82].

##### Infiltrative HCC

Infiltrative HCC is a rare type of HCC presenting as an ill-defined tumor that occupies multiple liver segments, an entire lobe, or the entire liver [86]. It is also known as diffuse, cirrhotomimetic HCC, or cirrhosis-like HCC [87]. Pathologically, it is characterized by the regional spread of diffuse, uniformly sized minute nodules resembling cirrhotic nodules without a dominant one [88]. Infiltrative HCC is commonly associated with portal vein thrombosis, high levels of alpha-fetoprotein (AFP), and very poor prognosis [86,87]. Due to the portal vein thrombosis, the typical enhancement pattern of HCC may be absent with only a slight enhancement in the arterial phase. In such cases, tumor thrombosis may be the leading sign of infiltrative HCC [89].

**Figure 19:**
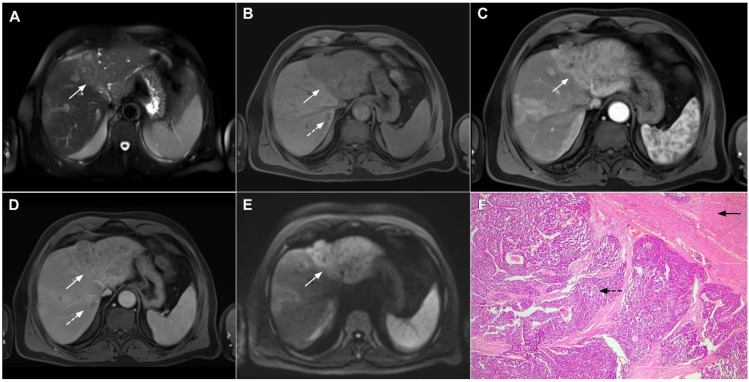
Infiltrative hepatocellular carcinoma in a 73-year-old man. The axial T2-weighted FS image (**A**) shows an ill-defined mass occupying the left liver lobe (*arrow*). Note the intratumoral biliary dilatation. The tumor (*arrow*) is hypointense in the plain T1-weighted image (**B**) and hypervascular in the arterial phase (**C**) with heterogeneous washout in the portal venous phase (**D**). In the DWI (**E**), the lesion (*arrow*) shows high signal intensity. Note also the small tumor nodule (*dashed arrow*) in the apical part of liver segment VII in (**B**,**D**). Hematoxylin and eosin (H&E) staining showed moderately-differentiated hepatocellular carcinoma (*dashed arrow*) and normal liver tissue next to the tumor (*arrow*); original magnification ×40 (**F**).

Taking into account the absence of typical vascular behavior and permeative ill-defined hypovascular appearance in images, infiltrative HCC may sometimes pose a diagnostic dilemma with mICC [90,91]. In this regard, patchy, miliary, or absent enhancement in the arterial phase with heterogeneous washout is characteristic for infiltrative HCC [86]. Nevertheless, the absence of washout should not exclude the diagnosis of infiltrative HCC [90]. In such cases, reticular appearance in the portal–venous phase, probably related to fibrosis and septa between minute tumor nodules, is highly suggestive of infiltrative HCC [92]. In the hepatobiliary phase, both tumors usually appear similarly as irregular hypointense lesions due to the lack of functional hepatocytes [86]. However, if cloud appearance consisting of a central hyperintensity and hypoattenuating rim is present then the diagnosis of mICC may be suggested [91]. Concerning appearance in T2-weighted images, Kim et al. have shown that infiltrative HCCs were relatively homogeneously mildly hyperintense in most cases, while mICC showed central areas of strong hyperintensity with or without areas of hypointensity reflecting its heterogeneous histopathological composition consisting of necrosis, mucin and fibrosis [91]. Furthermore, the appearance on DWI may be helpful in distinguishing between these entities, as mICC typically shows a targetoid appearance while infiltrative HCC has uniformly high signal intensity with low ADC values [92]. Other imaging features such as tumor thrombus is highly suggestive of infiltrative HCC, whereas mICC typically causes vascular encasement [91]. In addition, segmental biliary dilatation proximal to the tumor and capsular retraction favor the diagnosis of mICC, in contrast to infiltrative HCC, which may cause intratumoral biliary dilatation and contour bulging (Figure 19) [91].

#### 3.2.3. Combined Hepatocellular-Cholangiocarcinoma

Combined hepatocellular-cholangiocarcinoma (cHCC-CC) is a rare primary liver tumor exhibiting unequivocal characteristics of both HCC and mICC [93]. Although there are many theories regarding the origin of cHCC-CC, the most prominent hypothesis is that it derives from bipotent hepatic progenitor cells, which are capable of undergoing bidirectional differentiation into both hepatocytes and bile duct epithelial cells [94]. The presence of intimately mixed fully differentiated components of both HCC and ICC with the synchronous presence of transition zones is necessary for the definite histopathological diagnosis of cHCC-CC [94]. This is important for the differential diagnosis of collision tumors where both HCC and ICC are independently present in the same liver lobe [94].

**Figure 20:**
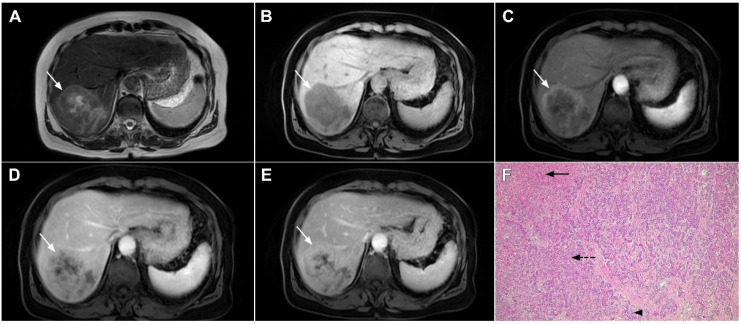
Combined hepatocellular–cholangiocarcinoma in a 59-year-old woman. In the T2-weighted image (**A**) a large tumor (*arrow*) with heterogeneously mildly increased signal intensity is seen in liver segment VII. The tumor (*arrow*) is hypointense in the plain T1-weighted image (**B**) with intense rim enhancement on the arterial phase (**C**), which gradually progresses centrally in the portal venous (**D**) and delayed phases (**E**). Hematoxylin and eosin (H&E) staining showed cells of hepatocellular differentiation (*dashed arrow*) and smaller zones of cholangiocellular differentiation (*arrowhead*). Normal liver parenchyma is also shown (*arrow*); original magnification ×40 (**F**).

The majority of studies indicate that cHCC-CC has intermediate clinical and radiological features between HCC and mICC with poorer prognosis compared to HCC and similar to ICC [95]. Imaging usually shows overlapping features between HCC and mICC [96]. Postcontrast behavior in cHCC-CC most commonly presents as peripheral enhancement in the early phase, with progressive central enhancement and peripheral washout in the delayed phase overlapping with images of mICC (Figure 20) [96]. Rarely, cHCC-CC may show classical HCC presentation with a diffuse arterial enhancement followed by washout, as was shown by Sammon et al. [97]. Concerning discordant results regarding the vascular behavior of cHCC-CC, Sanada et al. suggested that the presence of two distinct enhancement patterns in the same tumor favors the diagnosis of this rare entity [98]. Ancillary features characteristic of mICC such as capsular retraction and peripheral biliary dilatation are rarely present in cHCC-CC, thus suggesting the diagnosis of mICC [97]. In contrast, vascular encasement although specific to mICC may be also present in cHCC-CC [97]. Although distinguishing between cHCC-CC and mICC is hardly possible through preoperative imaging, since major hepatic resection with hilar node dissection remains the gold standard treatment for both entities, preoperative differentiation is not mandatory [99].

#### 3.2.4. Hemangioendothelioma

Hepatic epitheloid hemangioendotelioma (HEH) is an uncommon type of low-grade malignant hepatic neoplasm that originates from endothelial cells, occurring more commonly in females [100,101]. Clinical presentation is indistinguishable from other hepatic malignant tumors, ranging from no symptoms to liver failure [100]. Therapeutic options include resection or liver transplantation, as these tumors do not respond to chemo/radiotherapy [101].

**Figure 21:**
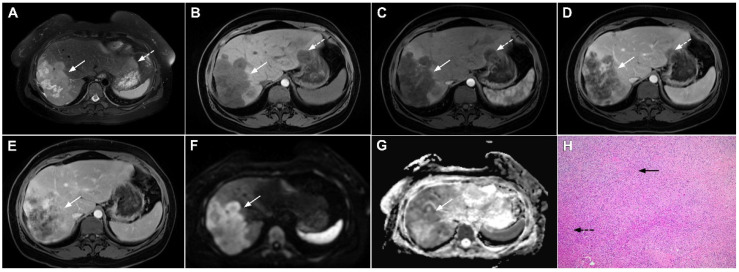
Hepatic hemangioendothelioma in a 44-year-old woman. The axial T2-weighted FS image (**A**) shows a heterogeneously hyperintense lesion (*arrow*) in liver segment VII, which is causing a slight capsular retraction. In the plain T1-weighted image (**B**), the tumor is hypointense. Another smaller lesion is also seen in liver segment II (*dashed arrow*). After administration of intravenous contrast media, there is only subtle perilesional enhancement in the arterial phase (**C**) with a gradual centripetal enhancement in the portal venous (**D**) and delayed phases (**E**). The tumor (*arrow*) shows high signal intensity in the DWI (**F**) with low ADC values on the periphery in the corresponding ADC map (**G**). Hematoxylin and eosin (H&E) staining showed epithelioid hemangioendothelioma (*arrows*). Normal liver parenchyma is also shown (*dashed arrow*); original magnification ×40 (**H**).

HEH may be of three different pathological types: single nodular, multifocal nodular, and diffuse type. The most common presentation is in the form of multiple, subcapsular nodes located in lesions that coalesce during the course of the disease [100]. Although multifocal nodular and diffuse types of HEH show characteristic radiological findings such as subcapsular localization, capsular retraction, and targetoid appearance on postcontrast study, the diagnosis of solitary HEH represents a diagnostic challenge [102]. Solitary lesion is the least frequent type of HEH and may closely resemble mICC [103]. Both lesions are heterogeneously hyperintense in T2-weighted images and hypointense in T1-weighted images [104]. With regard to tumor vascularity, Kim et al. have shown that solitary HEH usually shows minimal or rim-like enhancement in the early phase and delayed gradual fill-in, which is also a common vascular behavior of mICC (Figure 21). Moreover, both lesions may cause capsular retraction. In such difficult cases, the presence of the “lollipop sign” may suggest HEH [105]. This occurs when a well-defined liver nodule directly invades the vascular structure, resulting in its cut-off, which all together resembles the lollipop [105]. Furthermore, epidemiological data such as higher incidence in middle-aged females and normal values of CA 19-9 might be helpful in differential diagnosis, suggesting HEH [102]. On the other hand, if biliary dilatation is present adjacent to the tumor, the diagnosis of mICC is probable. The use of hepatobiliary contrast agents might be helpful in distinguishing between HEH and mICC, since mICC may show cloud-like appearance with central hyperintensity and peripheral hypointensity in the hepatobiliary phase, while it is rarely seen in HEH [102]. From the standpoint of treatment, the gold standard is radical surgical resection for both mICC and solitary HEH, thus preoperative differential diagnosis is not always necessary.

#### 3.2.5. Primary Hepatic Lymphoma

Primary hepatic lymphoma (PHL) is defined as a lymphatic malignancy limited to the liver without the involvement of other lymphatic tissues and organs for at least 6 months after appearance [106]. It is commonly seen in middle aged men, with a male to female ratio of 2.3:1 [107]. According to one hypothesis, it might be associated with chronic viral infections, particularly hepatitis C [107].

**Figure 22:**
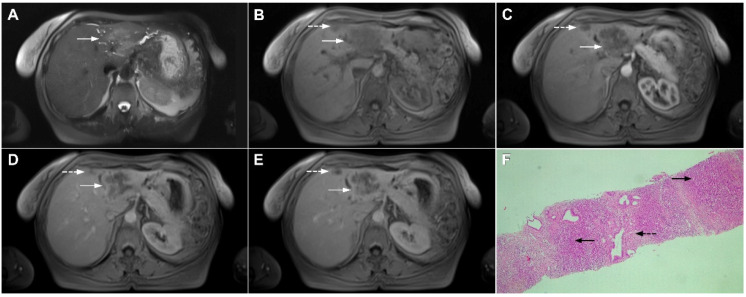
Primary hepatic lymphoma in a 72-year-old woman. The axial T2-weighted FS image (**A**) shows a moderately hyperintense lesion in liver segment II (*arrow*). Note also the biliary dilatation on the periphery of the lesion. In the plain T1-weighted image (**B**), the tumor (*arrow*) is hypointense with a slight enhancement in the arterial phase (**C**) and progressive central opacification in the portal venous (**D**) and delayed phases (**E**). A small satellite lesion is also seen (*dashed arrow*) in (**B**–**E**). Hematoxylin and eosin (H&E) staining showed non-Hodgkin liver lymphoma with T-cell histocyte-rich large B-cells (*arrows*). Remnants of biliary ductules are also shown (*dashed arrow*); original magnification ×40 (**F**).

Among the three morphological types, including single lesion, multiple lesions, and diffuse involvement of the liver, the single lesion is most commonly seen (55–60%), while the diffuse type is extremely rare [108]. Due to its unspecific radiological features, PHL may simulate mICC in images when it occurs as a single hepatic lesion. Both usually appear as ill-defined hypovascular tumors with subtle hyperintensity in T2-weighted images (Figure 22) [109]. Nevertheless, there are certain imaging characteristics that may facilitate the establishment of the correct diagnosis. Concerning growth pattern, Colagrande et al. have shown that PHL typically grows without deformation of blood and biliary vessels that pass through the lesion [109]. In contrast, mICC is commonly an infiltrative lesion, characterized by vessel displacement and/or constriction. With regard to appearance in DWI, PHL shows uniform diffusion restriction while mICC demonstrates hyperintensity only in the peripheral zone, known as the target sign [110]. In addition, PHL demonstrates significantly lower ADC values in comparison to mICC. One major difference is related to the delay phases where PHL usually remains hypointense whereas mICC shows progressive central enhancement [111]. Moreover, ancillary features commonly seen in mICC, such as capsular retraction and biliary dilatation, are usually not present in PHL [109].

## 4. Conclusions

In conclusion, we have described the key imaging features of mICC with an emphasis on the association between imaging presentation and clinicopathological features. In addition, we have provided a comprehensive overview of the benign and malignant lesions that may mimic mICC. Thorough interpretation of MRI examination data, including lesion appearance in T2-weighted images, DWI, and hepatobiliary phase in addition to the relative enhancement pattern, is mandatory for accurate lesion characterization. Moreover, ancillary features such as the shape of the lesion, the presence of biliary dilatation, and the relationship to liver capsules and vascular structures are very helpful in establishing a correct diagnosis. Considering the different treatment strategies for mICC and lesions simulating its appearance in images, it is of great clinical importance to differentiate mICC from its mimickers in order to avoid unnecessary surgery or delayed treatment of mICC with dismal prognosis.
